# Chlorogenic acid alleviates IPEC-J2 pyroptosis induced by deoxynivalenol by inhibiting activation of the NF-κB/NLRP3/caspase-1 pathway

**DOI:** 10.1186/s40104-024-01119-z

**Published:** 2024-12-02

**Authors:** Yanmei Xue, Fuchang Li, Rui Li, Xinru Zhang, Huijun Guo, Chunyang Wang

**Affiliations:** 1 Shandong Provincial Key Laboratory of Zoonoses, College of Animal Veterinary Medicine, Shandong Agricultural University, Tai’an, Shandong 271018 China; 2 College of Animal Science and Technology, Shandong Agricultural University, Tai’an, Shandong 271018 China

**Keywords:** Chlorogenic acid, Deoxynivalenol, IPEC-J2 cells, Pyroptosis, Signaling pathway

## Abstract

**Background:**

Deoxynivalenol (DON) is a mycotoxin that severely pollutes feed ingredients, and methods for reducing DON toxicity have become a significant research direction. Chlorogenic acid (CGA) is an active polyphenol found in some plants, which has anti-inflammatory and antioxidant properties and a protective effect on animal intestinal health. The effects of CGA on DON-induced pyroptosis in the intestinal porcine epithelial cell line-J2 (IPEC-J2) and its potential mechanism were explored in this study.

**Results:**

IPEC-J2 cells viability and membrane integrity were inversely correlated with DON concentration. Compared to those in the group treated with DON alone at 2,500 ng/mL, pretreatment with 80 μmol/L CGA for 4 h significantly improved cell viability (*P* < 0.01), and the alleviation of typical pyroptotic symptoms induced by DON were observed, including reduced cellular DNA fragmentation, decreased release of lactate dehydrogenase (LDH), normalized ROS levels, restoration of extracellular Ca^2+^ and K^+^ contents to normal levels (*P* < 0.01 ), as well as suppressed the enzyme activities of caspase-1 and caspase-4 (*P* < 0.01). Additionally, the mRNA expression levels of *TNF*,* MDP*,* NOD2*,* TLR4*,* ASC* and *GSDMD* were significantly improved (*P* < 0.01), while both mRNA and protein expression levels of NF-κB, NLRP3, caspase-1, IL-1β and IL-18 were significantly upregulated (*P* < 0.01) in the CGA + DON group, compare to those in the DON group.

**Conclusion:**

Pretreatment with 80 μmol/L CGA for 4 h effectively alleviated pyroptosis in IPEC-J2 cells induced by 2,500 ng/mL of DON through inhibiting activation of the NF-κB/ NLRP3/capase-1 pathway.

**Supplementary Information:**

The online version contains supplementary material available at 10.1186/s40104-024-01119-z.

## Introduction

Feed safety has a crucial impact on livestock health, and a series of problems caused by mycotoxin contamination in feed have been investigated in the context of animal husbandry and the feed industry [1, 2]. Among these mycotoxins, deoxynivalenol (DON) is a severely polluting mycotoxin that threatens the safety of human food and animal feed because of its high detection rate in various grains and its stable chemical properties [3, 4]. DON exhibits a range of toxic effects, with the intestine being its primary target organ [5]. After DON is ingested, the tight junction of intestine are disrupted, and simultaneously enhance the paracellular permeability of the enterocyte [6]. The intestinal tract serves as the first barrier to block its entrance to the body not only because of its location and function but also because it can mount an immune response to DON stimulation [7, 8]. Swine are the most sensitive animals to DON, and the intestinal porcine epithelial cell line-J2 (IPEC-J2) is a typical cell line used for studying intestinal injury. DON has proinflammatory and immunoregulatory effects on IPEC-J2 cells, inducing cellular damage such as oxidative damage, inflammation, and even apoptosis [9, 10].

Pyroptosis, which is a form of programmed cell death, is closely related to inflammation and is triggered by an imbalance in intracellular and extracellular homeostasis associated with innate immunity [11, 12]. Nucleotide oligomerization domain (NOD)-like receptor thermal protein domain-associated protein 3 (NLRP3) is a recognition receptor with an intracellular pattern that detects not only pathogenic microorganisms but also endogenous danger signals and certain metabolites [13]. After NLRP3 is activated, apoptosis-associated speck-like protein containing a caspase recruitment domain (ASC) is recruited. Subsequently, NLRP3 and the caspase-1 precursor assemble to form a multiprotein complex known as the NLRP3 inflammasome, also referred to as the caspase-1-dependent inflammasome. Caspase-1 can be activated by various stimuli, both in vivo and in vitro (such as certain extracellular ATP, RNA viruses, bacteria, and mycotoxins), through different pathways, thereby inducing pyroptosis [14, 15]. Swanson et al. [16] proposed a dual-signaling pattern of pyroptosis, which present in Additional file 1. The initiating signal (signal 1) involves the recognition of microbial ligands or cytokines by Toll-like receptors (TLRs), which subsequently activate the nuclear factor kappa-B (NF-κB) pathway, ultimately upregulating the levels of interleukin-1β (IL-1β) precursors and NLRP3 proteins. Moreover, muramyl dipeptide (MDP) and NOD-like receptor-2 (NOD 2) are also involved in the initiation step [17]. The activation signal (signal 2) promotes the assembly and activation of the NLRP3 inflammasome complex under pathological conditions, thereby leading to the activation of caspase-1 and overflow of IL-1β and interleukin-18 (IL-18), ultimately inducing pyroptosis [18]. Multiple studies have shown that DON can induce pyroptosis in various cell lines. However, the mode of initiation and activation is not clear [19, 20].

Given the severe contamination of DON in grains and feed materials, there is an urgent need to develop a novel type of feed additive capable of effectively reducing DON toxicity and enhancing the body’s immunity to alleviate the resulting damage. Owing to its weakly polar nature, DON poses challenges for removal via conventional physical adsorbents and chemical methods [21]. Therefore, nutritional regulation has emerged as a crucial research direction for mitigating DON toxicity [22–24]. Chlorogenic acid (CGA), which is a condensation of caffeic acid and quinic acid, is a phenylpropanoid that is primarily found in honeysuckle, Eucommia, coffee, and tea [25]. Multiple in vivo and in vitro experiments have shown that CGA has various biological activities, including anti-inflammatory, antioxidant, antibacterial, and intestinal protective effects [26–28]. CGA enhances the intestinal development and health of weaned piglets by inhibiting apoptosis and mucosal inflammation [29]. CGA has been reported to reduce the inflammatory response in ulcerative colitis induced by lipopolysaccharide (LPS) and ATP through inhibition of the NF-κB/NLRP3 pathway [30]. However, it remains unclear whether CGA alleviates DON-induced pyroptosis by inhibiting NLRP3 activation. This study aimed to analyze the effects of CGA on DON-induced pyroptosis and its potential mechanism, and then hope to provide a theoretical basement for the efficient prevention and treatment of intestinal pyroptosis induced by DON.

## Materials and methods

### Reagents

DON (C_15_H_20_O_6_, purity > 98%) was acquired from TripleBond Scientific Inc. (Guelph, Ontario, Canada), and a 1 mg/mL stock solution was prepared using acetonitrile, which was stored at 4 °C and diluted to different concentrations with serum-free DMEM high glucose culture medium in subsequent tests. CGA (C_16_H_18_O_9_, purity > 98%) was obtained from Macklin Company (Shanghai, China), and a 10 mmol/L stock solution was prepared using DMSO, which was stored at −80 °C and diluted to different concentrations with serum-free DMEM high glucose culture medium before use. The complete medium consisted of DMEM high glucose culture medium supplemented with 10% fetal bovine serum and a 1% penicillin–streptomycin mixture. The chemical reagents and detection kits utilized in this study are enumerated in Additional files 2 and 3, respectively.

### Cell culture

The IPEC-J2 cell line derived from porcine small intestine epithelial cells was provided by Shandong Agricultural University. The cells were cultured in complete medium and routinely maintained in a cell culture incubator (HealForce, Shanghai, China) at 37 °C with 5% carbon dioxide (CO_2_). Upon reaching the logarithmic growth phase, 1 mL of 0.25% pancreatic enzyme was added for cell digestion. After completion, the culture medium was transferred to a centrifuge tube and centrifuged at 1,000 r/min for 5 min. Following removal of the supernatant, complete medium was added to obtain a cell suspension, and then cell quantity and monolayer distribution were observed under an inverted microscope.

### Treatments

For the purpose of this study, IPEC-J2 cells were categorized into the following treatment groups: (I) the control group, without any treatment; (II) the screening group for a challenge dose of DON, treated with different concentrations of DON (500, 1,000, 1,500, 2,000, 2,500 and 3,000 ng/mL) for 12, 24 or 48 h; (III) the DON group, treated with DON at a concentration of 2,500 ng/mL for 24 h, based on the results of challenge dose screening; (IV) the screening group for a protective dose of CGA, pretreated with various concentrations of CGA (0, 10, 20, 40, 60, 80 and 100 μmol/L) for 4 h followed by exposure to DON at a concentration of 2,500 ng/mL for 24 h; (V) the CGA group, with CGA treatment at a concentration of 80 μmol/L for 4 h, based on the results of protective dose screening; and (VI) the CGA + DON group, pretreated with CGA at a concentration of 80 μmol/L for 4 h followed by exposure to DON at a concentration of 2,500 ng/mL for 24 h.

### CCK-8

Each well of a 96-well plate was inoculated with 100 μL of cell suspension at a density of 2 × 10^4^ cells/mL, and cultured until cellular adhesion occurred. Following different treatments, a 10 μL of CCK-8 solution was added into each well and incubated at 37 °C for 2 h. Next, the absorbance at 450 nm was subsequently quantified by an automated microplate reader (BioTek, Vermont, USA), followed by calculation of cell viability based on the instructions of the CCK-8 assay kit.

### MTT

Each well of a 96-well plate was inoculated with 180 μL of cell suspension at a density of 5 × 10^4^ cells/mL, and cultured until cellular adhesion occurred. Following various treatments, 10 μL of MTT solution was added to each well and incubated for 4 h. Subsequently, the supernatant was removed and replaced with 110 μL of formazan solution, which was gently shaken for 10 min. An automated microplate reader (BioTek, Vermont, USA) was employed to detect the absorbance at 490 nm, which was subsequently used for calculate the cell calculation mortality based on the instruction of MTT assay kit.

### Lactate dehydrogenase (LDH) activity assay

Cells at a density of 1 × 10^4^ cells/well were inoculated into a 96-well culture plate and incubated until they adhered to the wall. Following different treatments, 150 μL of LDH release reagent was added, and the mixture was incubated for 1 h. Following the guidance of the LDH assay kit, the supernatant was collected and incubated with 60 μL of LDH working solution for a duration of 30 min. The absorbance at 490 nm was measured using an automated microplate reader (BioTek, Vermont, USA).

### Enzyme-linked immunosorbent assay (ELISA)

A cell suspension containing 2 × 10^5^ cells/well was inoculated into a 6-well plate and incubated until they adhered to the wall. Following various treatments, pancreatic enzymes were used to digest the cells, after which the cell suspension was collected and subjected to ultrasonic lysis in an ice bath. The supernatant was collected after centrifugation and processed following the instructions provided by the ELISA kits. The absorbance at 450 nm was subsequently measured by an automatic microplate reader (BioTek, Vermont, USA).

### Caspase-1/4 activity assay

A cell suspension at a density of 2 × 10^5^ cells/well was distributed into a 6-well plate and incubated to adhere. After undergoing diverse treatments, the cells were harvested and lysed at 4 °C. Subsequently, the supernatants were co-incubated with the substrates of caspase-1 (Ac-YVAD-pNA) or caspase-4 (Ac-LEVD-pNA) at 37 °C for 2 h. Ultimately, the yellow* p*-nitroaniline (*p*NA) was quantitatively assessed by a UV spectrophotometer (Shimadzu, Tokyo, Japan), and the enzyme activity of caspase-1/4 were measured following the recommendations of the caspase-1/4 activity assay kit.

### Colorimetry

Colorimetric method was used to detect the extracellular concentrations of Ca^2+^ and K^+^. A cell suspension at a density of 2 × 10^5^ cells/well was distributed into a 6-well plate and incubated to adhere. After undergoing diverse treatments, the cell culture supernatant was collected and centrifuged at 12,000 r/min for 10 min. Subsequently, the resulting supernatant was transferred to a fresh centrifuge tube. Following the instructions of cation detection kits, the corresponding colorimetric reagents were added to the supernatant, and then the concentrations of Ca^2+^ and K^+^ were calculated based on the values of OD_610nm_ and OD_440nm_ measured via a UV spectrophotometer (Shimadzu, Tokyo, Japan).

### Lyso-Tracker Red (LTR)

An LTR assay kit was used to detect intracellular lysosomes. Cells at a density of 2 × 10^5^ cells/well were inoculated onto a cell slide placed in a 6-well plate and cultured until they adhered to the surface. After the cells were treated with various concentrations of DON, they were co-cultured with LTR dye working solution for 1 h. Finally, images were captured under a fluorescence microscope (NIKON, Tokyo, Japan) and analyzed using Image J 1.54f (NIH, Maryland, USA).

### Scanning electron microscopy (SEM)

Cells at a density of 2 × 10^5^ cells/well were inoculated onto the cell slides and then treated with different concentrations of DON for 24 h. The slides were gently rinsed with PBS buffer and fixed with an electron microscope fixing solution at room temperature in the dark for 30 min. Subsequently, a series of steps were performed, including rinsing with 0.1 mol/L phosphate buffer, gradient dehydration with increasing concentrations of ethanol, replacement with isoamyl acetate, drying at the CO_2_ critical point, and gold plating with an ion sputter coater. Finally, the images were observed and captured using an SU8100 SEM (Hitachi, Japan).

### Reactive oxygen species (ROS)

Dichlorofluorescin diacetate (DCFH-DA) was used to measure the level of intracellular ROS. A cell suspension with a density of 2 × 10^5^ cells/well was evenly spread in a confocal dish. Following various treatments, 1 mL of 10 μmol/L DCFH-DA working solution was added to each confocal dish, which was subsequently incubated at 37 °C for 20 min in the dark. The fluorescence images were subsequently observed using a dragonfly confocal microscope (Andor, Belfast, Northern Ireland), and the fluorescence intensity was analyzed using Image J 1.54f (NIH, Maryland, USA). 1 mL of 10 μmol/L DCFH-DA working solution (MedChemExpress, USA) was added to each confocal dish and incubated for 20 min. Subsequently, 1 mL of the cell culture medium was added, and images were captured using a confocal laser microscope.

### Acridine orange/ethidium bromide (AO/EB) dual staining

An AO/EB dual-dye kit was used to observe the nuclear state. The cells were spread into small confocal dishes at a density of 2 × 10^5^ cells/well and incubated to cell adhesion. After various treatments, the cells were resuspended in 500 μL staining buffer and then sequentially dyed with 5 μL each of AO and EB dye solutions, followed by incubation at 4 °C for 10–20 min. The images were observed using a dragonfly confocal microscope, and the fluorescence intensity was analyzed using Image J 1.54f.

### Terminal deoxynucleotidyl transferase–mediated dUTP-biotin nick end labeling assay (TUNEL)

A TUNEL assay kit was used for the detection of DNA fragments generated during apoptosis or pyroptosis. The cells were inoculated at a density of 2 × 10^5^ cells/well on the cell slides and cultured until they reached adhesion. After various treatments, the cells were fixed with 4% paraformaldehyde for 30 min. Subsequently, the cells were incubated with 100 μL of TUNEL reaction solution at 37 °C for 60 min, after which the reaction was terminated. The images were observed using a confocal fluorescence microscope (NIKON, Tokyo, Japan).

### Quantitative real-time PCR (qRT-PCR)

The cells were inoculated into a 6-well culture plate at a density of 2 × 10^5^ cells/well and cultured until they reached adhesion. Following different treatments, total RNA was extracted using TRIzol and reverse-transcribed into cDNA according to the instructions provided by the PrimeScript® RT Reagent kit. The real-time PCR assay was conducted using an ABI 7500 real-time PCR system (Waltham, USA) in a total reaction volume of 20 μL. The PCR conditions and calculation method for determining relative mRNA expression levels based on the 2^−ΔΔCt^ method were described in previous study [31]. All primers, including the reference gene* GAPDH*, were synthesized by Sangon Biotech (Shanghai, China) and were listed in Additional file 4.

### Western blotting

The cells were inoculated into a 6-well culture plate at a density of 2 × 10^5^ cells/well. Following different treatments, the cells were lysed in RIPA buffer supplemented with PMSF to obtain the total protein. Subsequently, equal amounts of denatured proteins separated by SDS‒PAGE were transferred onto a polyvinylidene fluoride (PVDF) membrane. After blocking with TBST for 2 h, the membranes were incubated overnight at 4 °C with the primary antibody followed by a 2-h incubation at room temperature with the secondary antibody. Finally, after image development using the FX7 Vilber Lourmat imaging system (Fusion, France), the optical densities of the target protein bands were evaluated using Image J 1.54f. β-Actin was concurrently tested as an internal reference in this study. The antibody information for IL-1β, IL-18, NlLRP3, and caspase-1 utilized in Western blot assay is provided in Additional file 5.

### Immunofluorescence (IF)

IF assay was used to detect the expression of NF-κB and caspase-1. Cells at a density of 2 × 10^5^ cells/well were inoculated on the cell slides and cultured until they reached adhesion. Following various treatments, the cells were fixed with 4% paraformaldehyde. After treatment with Triton X-100 and 5% bovine serum albumin, the slides were incubated with primary antibodies at 4 °C overnight and then with corresponding secondary antibodies labeled with HRP at room temperature for 1 h. Subsequently, the cell nucleus were stained to blue using DAPI and the photographs were captured using a confocal fluorescence microscope (NIKON, Japan). The expression of NF-κB and caspase-1 was detected using an IF assay, and their antibody information is provided in Additional file 5.

### Statistical analysis

The results were prep-processed using EXCEL followed by one-way and two-factor ANOVA using the GLM program in SAS version 9.4 (North Carolina, USA). Duncan’s multivariate comparison tests were performed to determine the significance of comparisons between means. All analysis results are expressed as the mean ± SD, where *P* ≤ 0.05 indicates a significant difference, and *P* ≤ 0.01 indicates an extremely significant difference. Finally, GraphPad Prism version 8.0 for Windows (California, USA) was used to generate the figures.

## Results

### DON induces pyroptosis

**Fig. 1:**
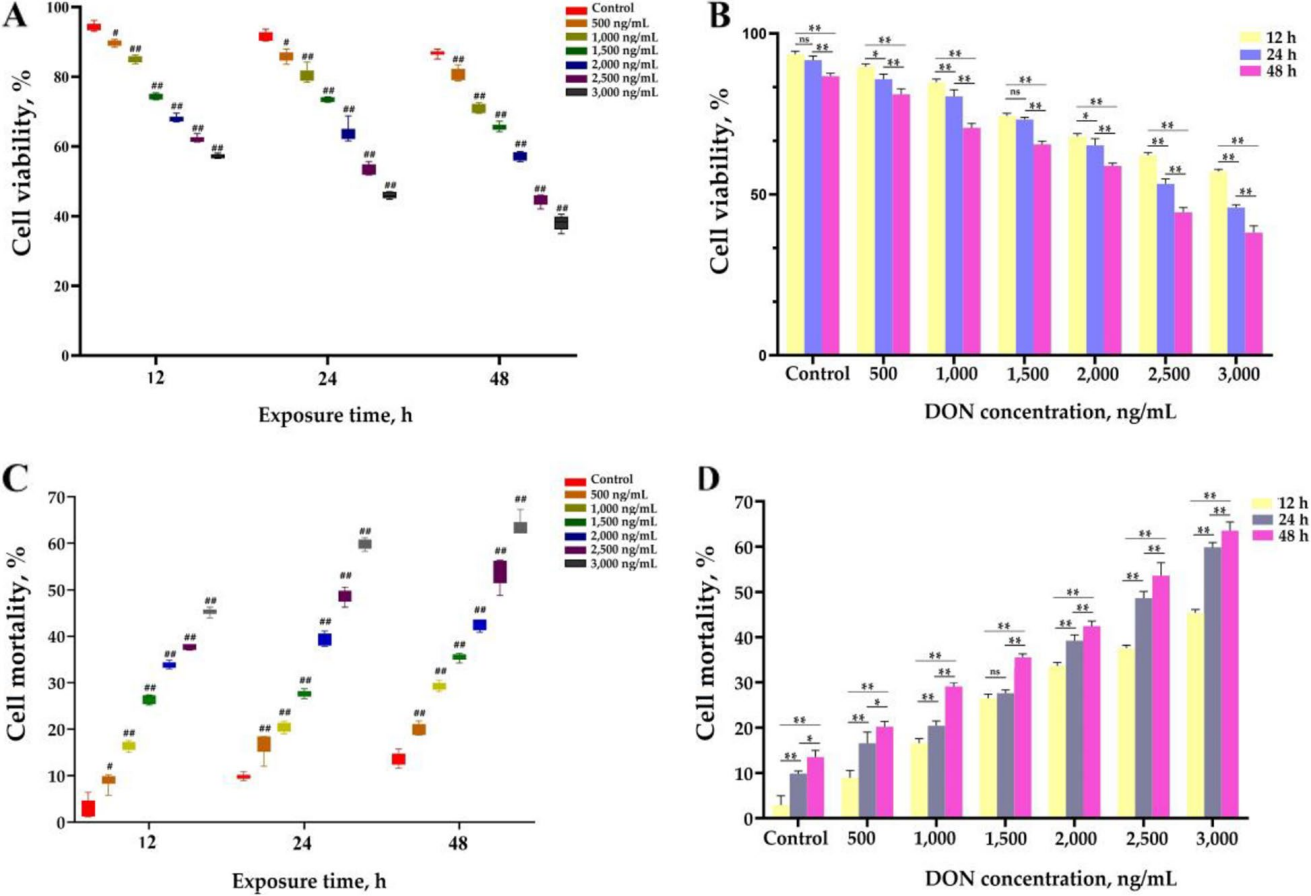
Effects of DON exposure at different concentrations and duration on the viability and mortality of IPEC-J2 cells (mean ± SD, *n* = 6). **A** and **B** Cell viability influenced by DON exposure time and concentration. **C** and **D** Cell mortality influenced by DON exposure time and concentration. Compared with the control group, ## and # represent *P* < 0.01 and* P* < 0.05 respectively. ns, * and ** indicate *P* > 0.05, *P* < 0.05 and *P* < 0.01, respectively. DON: deoxynivalenol

The first step of this study was to screen the concentration and duration of DON exposure for subsequent investigations, utilizing cell viability, membrane integrity, and the levels of several pyroptosis factors as evaluation criteria. The viability and mortality of IPEC-J2 cells exposed to DON concentrations ranging from 500 to 3,000 ng/mL were assessed using CCK-8 and MTT assays. The CCK-8 assay results demonstrated a significant inverse correlation between cell viabilit**y** and the DON concentration gradient (*P* < 0.01), with lower cell viability (*P* < 0.05) observed under the same concentration as the culture time increased (Fig. 1A and B). Additionally, MTT assay data revealed a positive correlation between cell mortality and the concentration gradient of DON (*P* < 0.01), accompanied by a positive correlation between cell mortality and the duration time under the same concentration (*P* < 0.05) (Fig. 1C and D). When the concentration of DON exceeded 2,500 ng/mL, the cell mortality surpassed 40%. Based on these results, a 24-h exposure time to DON was chosen for subsequent studies.

**Fig. 2:**
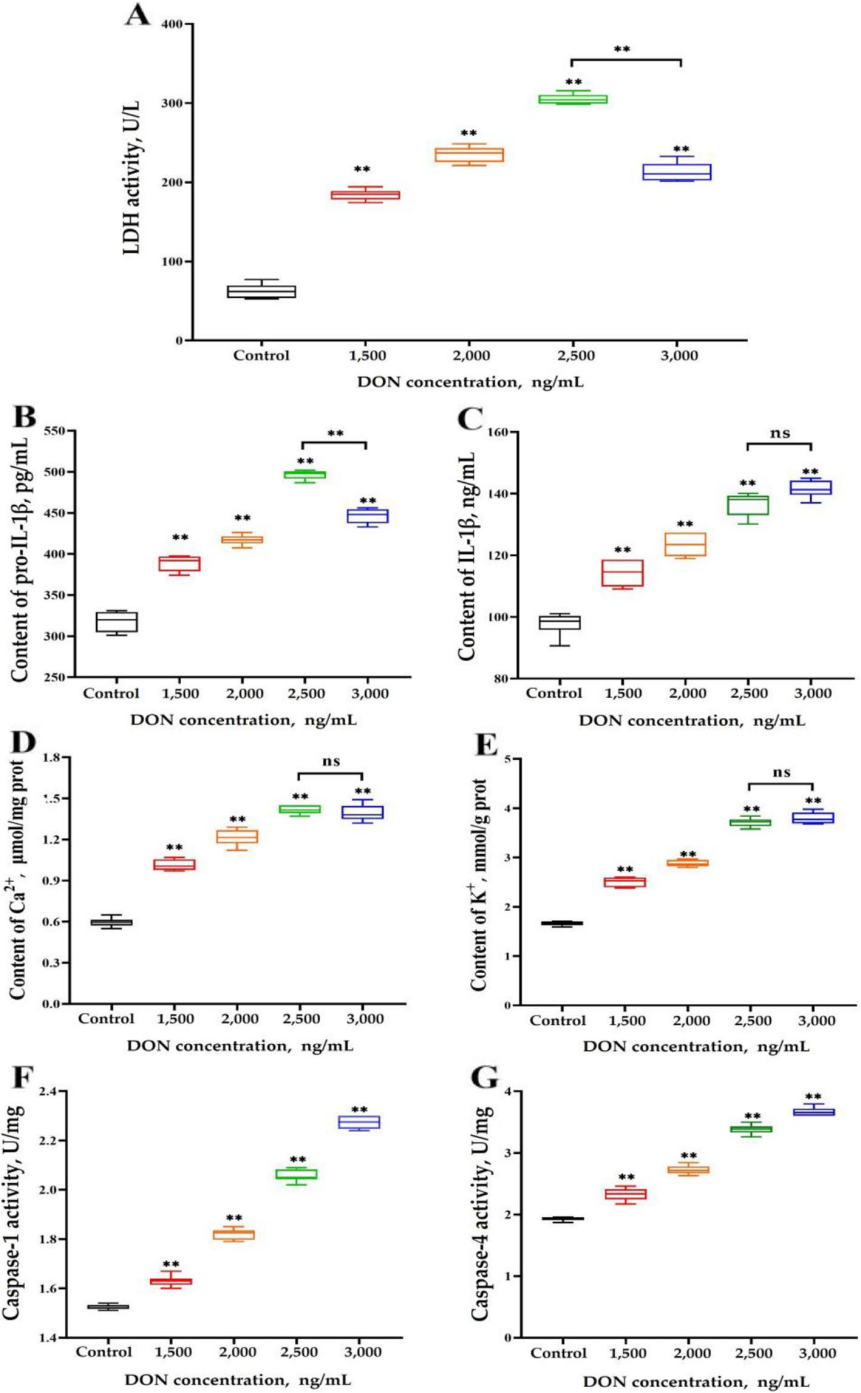
Effects of DON at different concentrations on several pyroptosis-related factors (mean ± SD, *n* = 6). **A** LDH activity. **B** and **C** Contents of pro-IL-1β and IL-1β. **D** and **E** Contents of extracellular Ca^2+^ and K^+^. **F** and **G** Enzyme activity of caspase-1 and caspase-4. Significance was compared with the control group unless otherwise indicated, * indicates *P* < 0.05 and ** indicates* P* < 0.01. ns indicates *P* > 0.05

The effects of DON concentrations ranging from 1,500 to 3,000 ng/mL on several pyroptosis-related factors are displayed in Fig. 2. Compared with that in the control group, LDH activity and pro-IL-1β content significantly increased in all gradients of DON (*P *< 0.01). Interestingly, there was a significant decrease in LDH activity and pro-IL-1β content when the concentration of DON was 3,000 ng/mL compared to 2,500 ng/mL (*P* < 0.01; Fig. 2A and B). Additionally, the levels of IL-1β, Ca^2+^, K^+^, caspase-1 and caspase-4 were significantly greater in all DON groups than those in the control group (*P* < 0.01), indicating a direct correlation with DON concentration (Fig. 2C–G). It is worth noting that there were no significant differences observed in the levels of IL-1β and Ca^2+^/K^+^ between DON exposures at 3,000 and 2,500 ng/mL (*P* > 0.05).

**Fig. 3:**
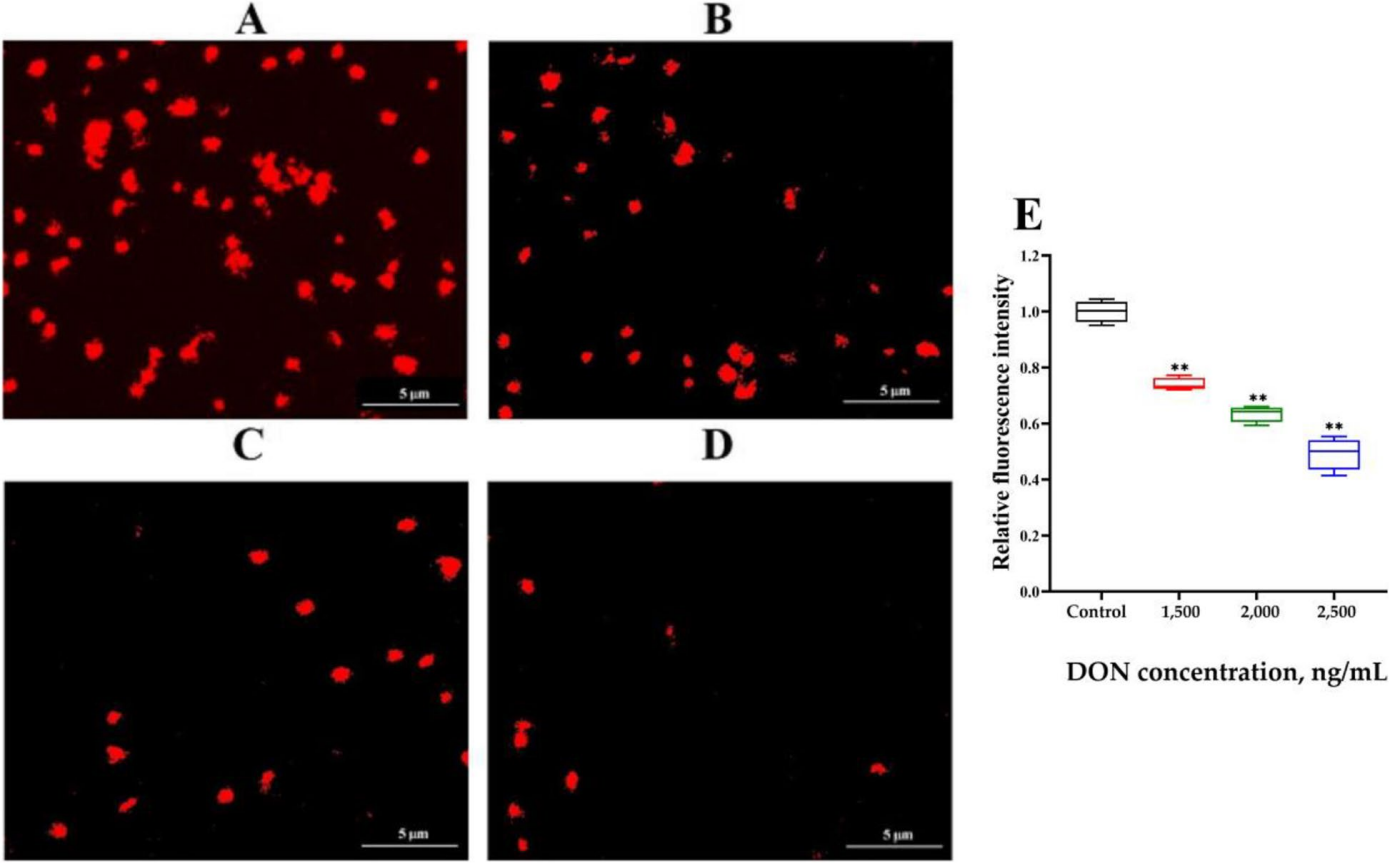
Effects of different concentrations of DON on lysosomes in IPEC-J2 cells detected by the Lyso-Tracker Red (mean ± SD, *n* = 3). Typical fluorescent staining of lysosomes at DON concentrations of (**A**) 0, (**B**) 1,500, (**C**) 2,000, and (**D**) 2,500 ng/mL. Scale bar is 5 μm. **E** Red fluorescence intensity of lysosomes. Significance was compared with the control group, and ** indicates* P* < 0.01

**Fig. 4:**
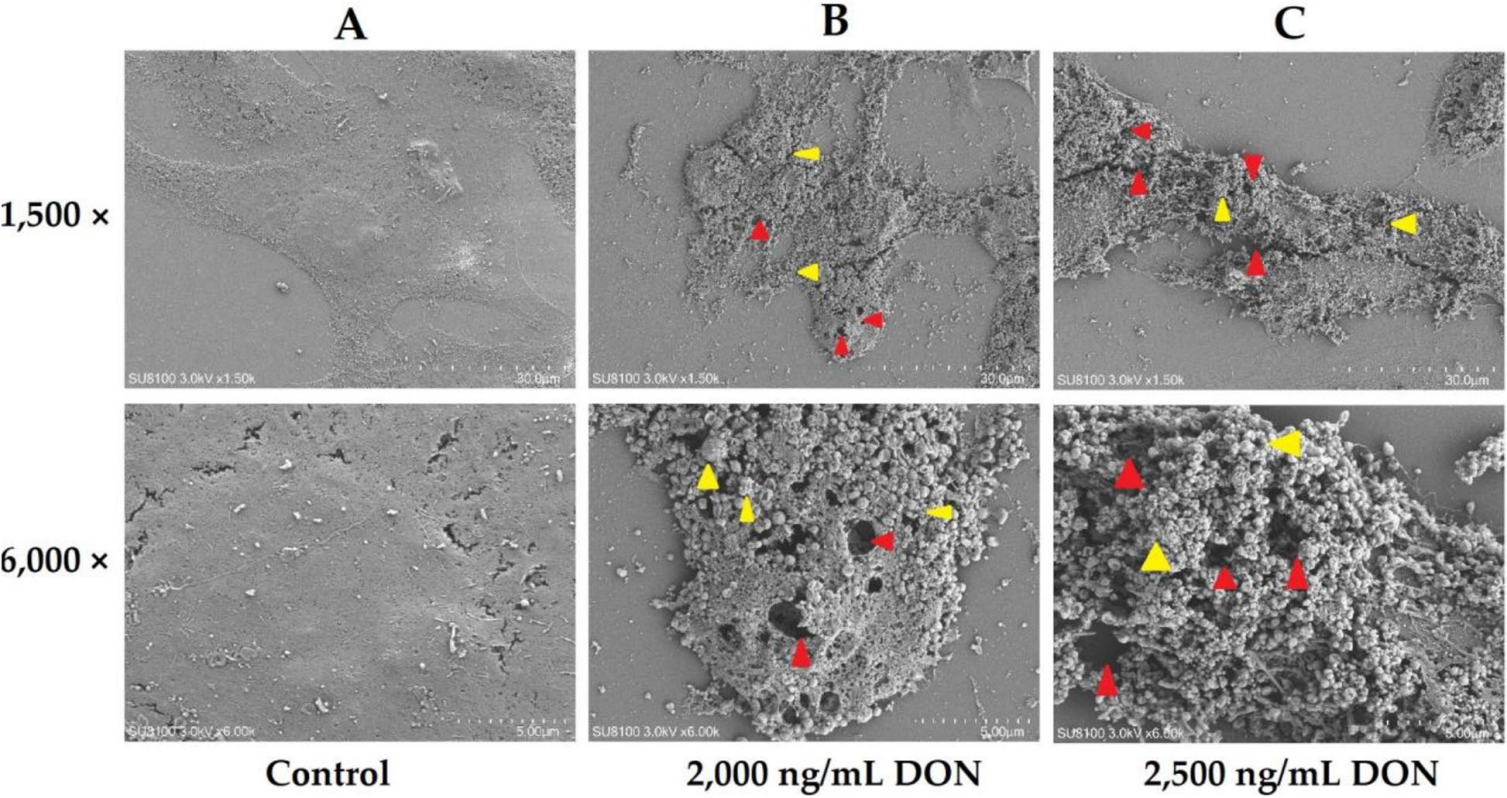
Effects of DON exposure on the ultrastructure of IPEC-J2 cells observed via scanning electron microscopy (*n* = 3). DON exposure concentrations of (**A**) 0, (**B**) 2,000, and (**C**) 2,500 ng/mL. Red arrows indicate pores, whereas yellow arrows indicate vesicles formed by cell protrusions. 1,500× and 6,000× represent the magnification of electron microscopy

The LTR results (Fig. 3) revealed a significant decrease in the fluorescence intensity of intracellular lysosomes (*P* < 0.05) caused by DON exposure, which was inversely proportional to the concentration of DON, indicating that the activity of intracellular lysosomes were destroyed by DON exposure. The SEM results (Fig. 4) revealed that when the DON concentration was 2,000 ng/mL, the cells swelled, and vesicles formed owing to the presence of numerous cell protrusions. The cell membrane was extensively damaged with numerous pores of varying sizes observed, which is consistent with pyroptosis features. The cell membrane in the group treated with DON at 2,500 ng/mL presented more severe damage, accompanied by more holes and vesicles (Fig. 4C). Therefore, a concentration of 2,500 ng/mL DON was used for subsequent experiments.

### CGA alleviates injury to IPEC-J2 cells induced by DON

**Fig. 5:**
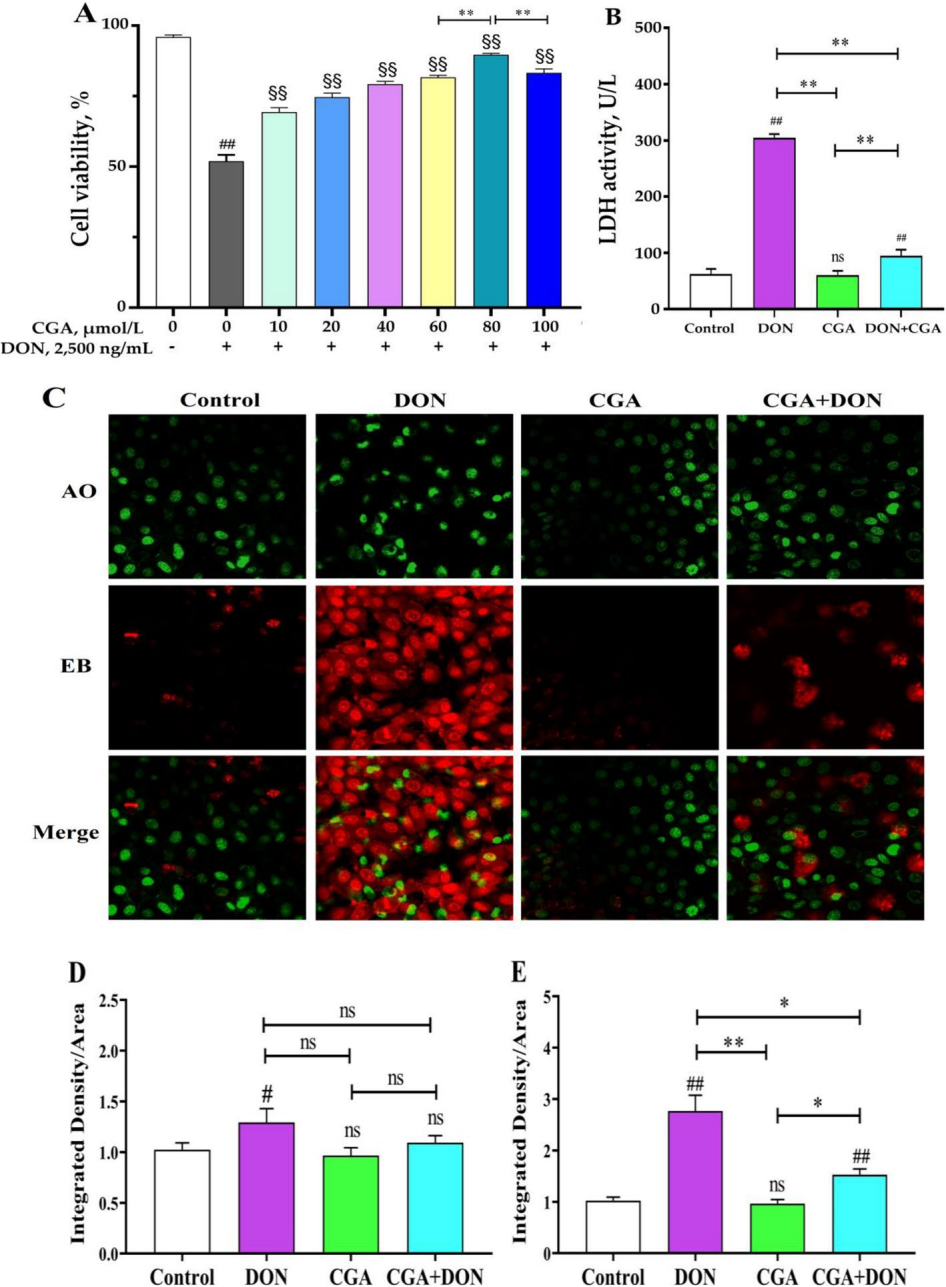
CGA alleviates the breakdown of IPEC-J2 cells induced by DON. **A** Cell viability of gradient CGA pretreatment against 2,500 ng/mL. **B** LDH activity. **C** Photos dyed by acridine orange and ethidium bromide, and green (**D**) and red (**E**) fluorescence intensity (mean ± SD, *n* = 6). Compared with the control group, ## and # represent *P* < 0.01 and *P* < 0.05 respectively. Compared with DON alone group, §§represents *P* < 0.01. Compare with other group, ** and * represent *P* < 0.01 and *P* < 0.05 respectively. ns represents *P* > 0.05

After pretreatment with CGA at concentrations ranging from 0 to 100 μmol/L for 4 h, IPEC-J2 cells were further exposed to 2,500 ng/mL DON for 24 h, and the optimal inhibitory concentration of CGA against 2,500 ng/mL DON was evaluated via the CCK-8 assay. The results (Fig. 5A) demonstrated that, compared with the DON alone group, varying concentrations of CGA effectively increased the cell viability treated with 2,500 ng/mL DON (*P* < 0.05), showing an initial increase but then subsequently decreased. Among them, the highest level of cell viability was observed upon the addition of 80 μmol/L CGA (*P* < 0.01). The LDH activity in the DON group was markedly elevated (*P* < 0.01) when compared to both the control group and the CGA group (Fig. 5B). Although the LDH activity in the CGA + DON group was significantly greater than that in the CGA group (*P* < 0.01), it was substantially lower than that observed in the DON group (*P* < 0.01).

The apoptosis of IPEC-J2 cells was observed under a fluorescence microscope using AO/EB double staining, and representative images are shown in Fig. 5C. The AO staining images revealed that the cell nuclei in the CGA and CGA + DON groups exhibited intact morphologies with distinct boundaries, whereas those in the DON group presented signs of damage. Furthermore, EO-stained images revealed a significant increase in both the number and intensity of red-positive apoptotic cells in the DON group compared with those in the other three groups. The results of the EO staining analysis (Fig. 5D) revealed a substantial increase (*P* < 0.01) in fluorescence intensity in the DON group compared with that of the control and the CGA groups. Although the fluorescence intensity of the DON + CGA group was significantly greater than that of the control group (*P* < 0.01), it was significantly lower than that of the DON group (*P* < 0.01), indicating that CGA can alleviate DON-induced apoptosis.

### CGA relieves IPEC-J2 pyroptosis induced by DON

**Fig. 6:**
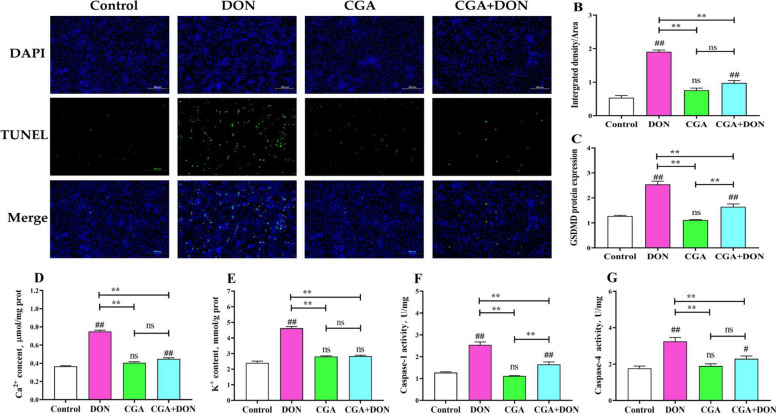
CGA alleviates DON-induced pyroptosis in IPEC-J2 cells (mean ± SD, *n* = 6). **A** and **B** Typical TUNEL dying photograph and fluorescence intensity (mean ± SD, *n* = 6). Scale bar is 100 μm. **C** mRNA level of *GSDMD*. **D** and **E** Extracellular contents of Ca^2+^ and K^+^. **F** and **G** Enzyme activity of caspase-1 and caspase-4. ns indicates *P* > 0.05. Compared with the control group, ## and # represent *P* < 0.01 and* P* < 0.05 respectively, and compared with the other group, ** and * represent *P* < 0.01 and* P* < 0.05 respectively. GSDMD: gasdermin D

Unlike apoptosis, the most prominent features of pyroptosis include degradation of chromosomal DNA, loss of cell membrane integrity, and overflow of IL-1β and IL-18 from the cytoplasm. Positive green fluorescent nuclei stained by TUNEL represent dead IPEC-J2 cells with broken DNA. The intensity of green fluorescence in the DON group was noticeably greater than (*P* < 0.01) that in the other three groups (Fig. 6A and B). The fluorescence intensity in the CGA + DON group was significantly lower (*P* < 0.01) than that in the DON group, but higher than that in the control group (*P* < 0.01), indicating that CGA can reverse the DNA damage induced by DON. Compared with those in the control and CGA groups, DON addition significantly increased the mRNA level of *GSDMD* (*P* < 0.01), extracellular content of Ca^2+^ (*P* < 0.01) and K^+^ (*P* < 0.01), as well as the enzyme activities of caspase-1 (*P* < 0.01) and caspase-4 (*P* < 0.01). While, these changes were significantly reduced (*P* < 0.01) after pretreating with CGA compared to those in the DON group (Fig. 6C–G).

**Fig. 7:**
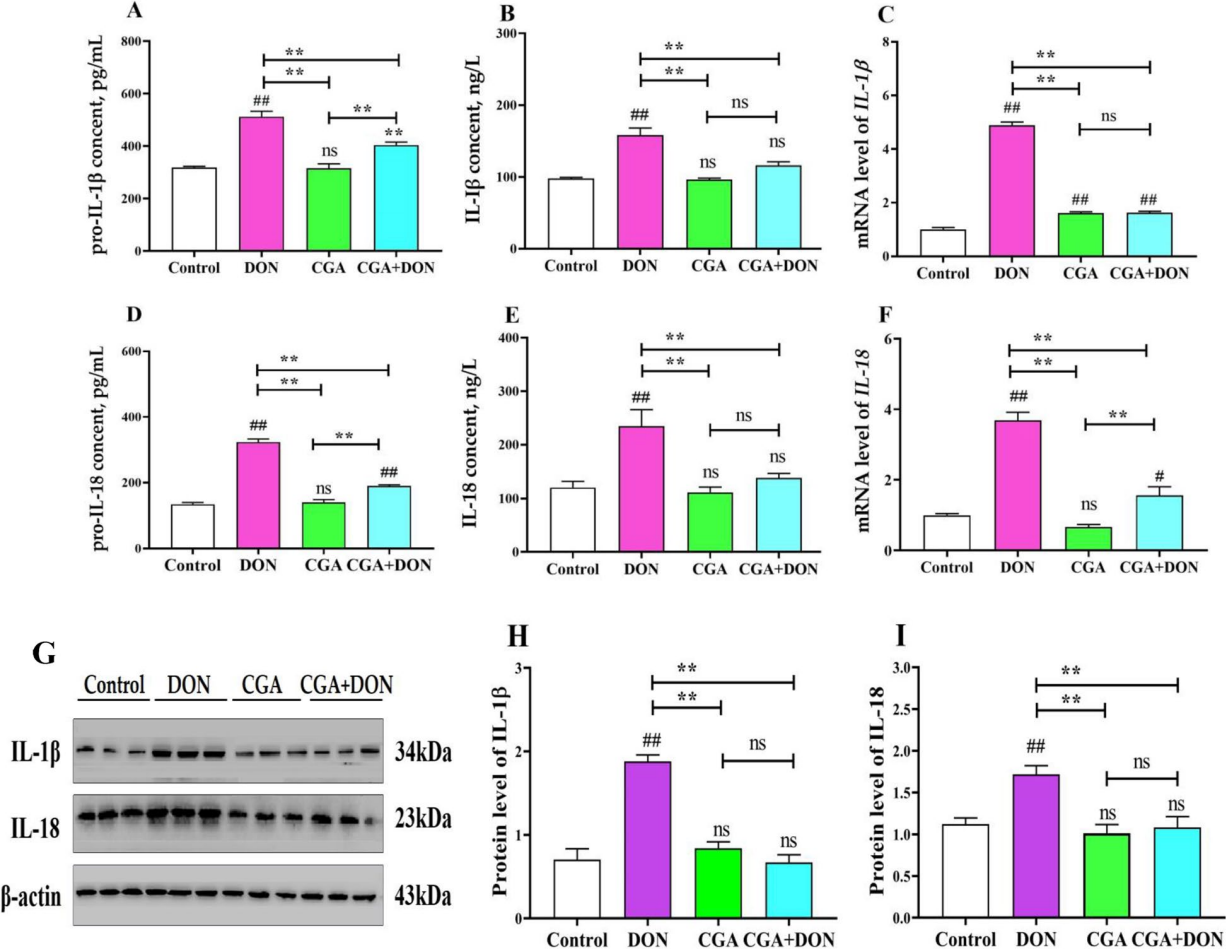
Effects of CGA and DON on key proinflammatory factors related to pyroptosis (mean ± SD, *n* = 3). Contents of (**A**) pro-IL-1β, (**B)** IL-1β, (**D)** pro-IL-18, and (**E**) IL-18. mRNA levels of (**C**) *IL-1β* and (**F**) *IL-18.* **G** Protein bands, and relative protein expression levels of (**H**) IL-1β and (**I**) IL-18. ns indicates *P* > 0.05. Compared with the control group, ## and # represent *P* < 0.01 and *P* < 0.05 respectively, and compared with the other group, ** and * represent *P* < 0.01 and *P* < 0.05 respectively

ELISA, qRT‒PCR and Western blotting were used in this study to assess changes in key proinflammatory factors related to pyroptosis (Fig. 7). Compared with those in the control group, DON exposure significantly increased the contents of pro-IL-1β (*P* < 0.01), IL-1β (*P* < 0.01), pro-IL-18 (*P* < 0.01), and IL-18 (*P* < 0.05), as well as the mRNA and protein expression levels of IL-1β (*P* < 0.01) and IL-18 (*P* < 0.01). However, compared to the DON group, these data were significantly decreased (*P* < 0.01) in the CGA + DON group. These findings suggest that DON can stimulate the release of intracellular cations and proinflammatory elements, thereby inducing pyroptosis.

### CGA inhibits DON-induced pyroptosis via a dual-signaling pathway

**Fig. 8:**
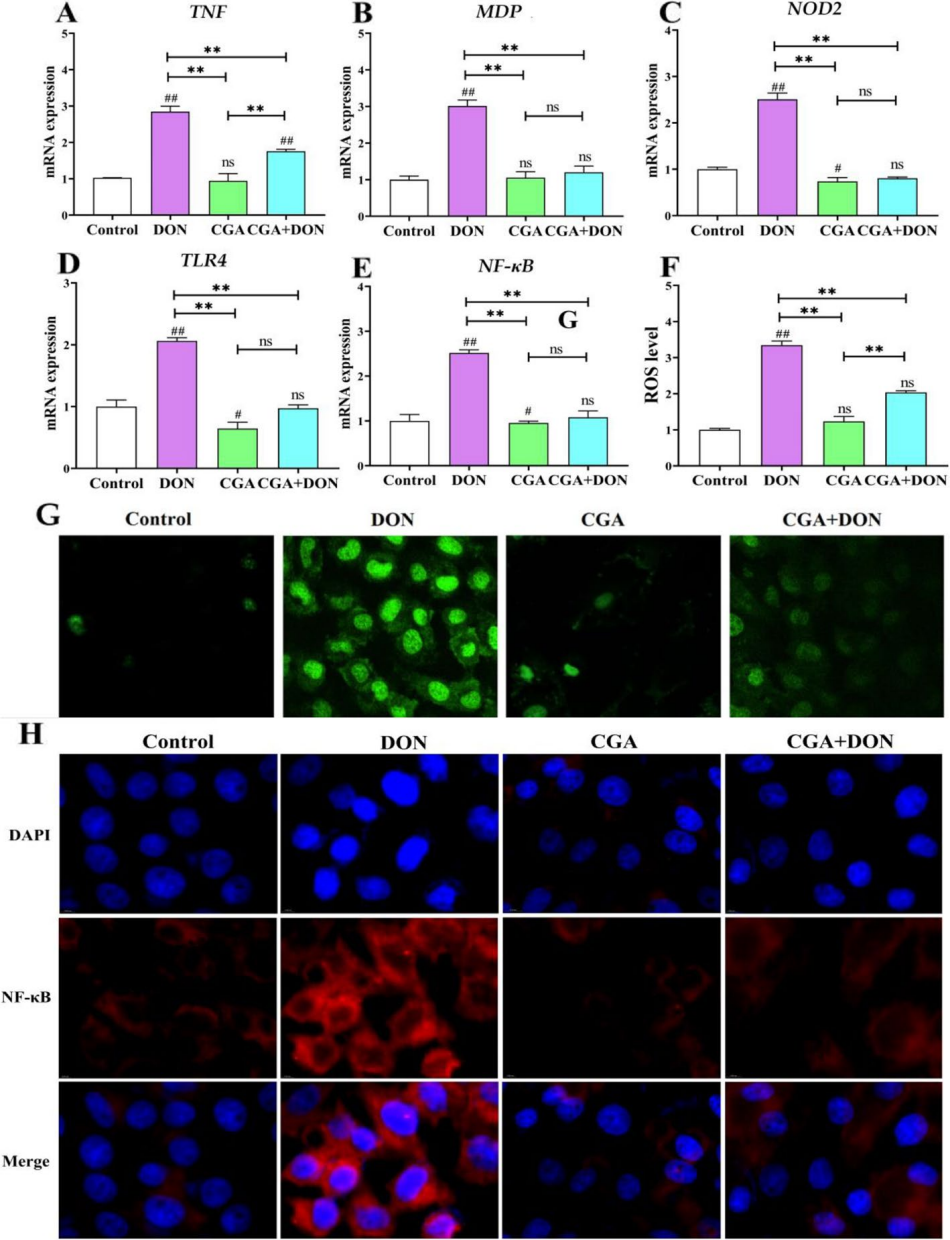
Effects of CGA and DON on initiation signaling factors related to pyroptosis. **A–E** mRNA levels of *TNF*, *MDP*, *NOD2*, *TLR4*, and *NF-κB* (mean ± SD, *n* = 6). **F** and **G** ROS level and fluorescence photo (mean ± SD, *n* = 3). **H** Visualized fluorescence photos of NF-κB (*n* = 3). Compared with the control group, ## and # represent *P* < 0.01 and *P* < 0.05 respectively, and compared with the other group, ** and * represent *P* < 0.01 and *P* < 0.05 respectively. ns indicates *P* > 0.05

To further determine the inhibitory effect of CGA on DON-induced pyroptosis, this study examined the mRNA levels of key signaling factors involved in the priming and activation of pyroptosis pathways. Compared with those in the control and CGA groups, DON exposure significantly increased the mRNA expression levels of *TNF*, *MDP*, *NOD*2, *TLR4*, and *NF-κB* (*P* < 0.01), which are related to pyroptosis priming signaling (Fig. 8A–E). However, the mRNA expression levels of *TNF*, *MDP*, *NOD2*, *TLR4* and *NF-κB* in the CGA + DON group exhibited a significant decrease compared to those in the DON group (*P* < 0.01). The DCFH-DA assay results (Fig. 8F and G) revealed that DON exposure markedly increase the levels of ROS compared with that in the control group (*P* < 0.01). Whereas, the ROS level in the CGA + DON group was lighter than that in the DON group (*P* < 0.01), yet significantly greater than that in the CGA group (*P* < 0.01). The IF images illustrated that the fluorescence intensity of NF-κB in the DON group was significantly greater than that in the other three groups (Fig. 8H), indicating that DON exposure promoted the expression of NF-κB and that the addition of CGA minimized these changes.

**Fig. 9:**
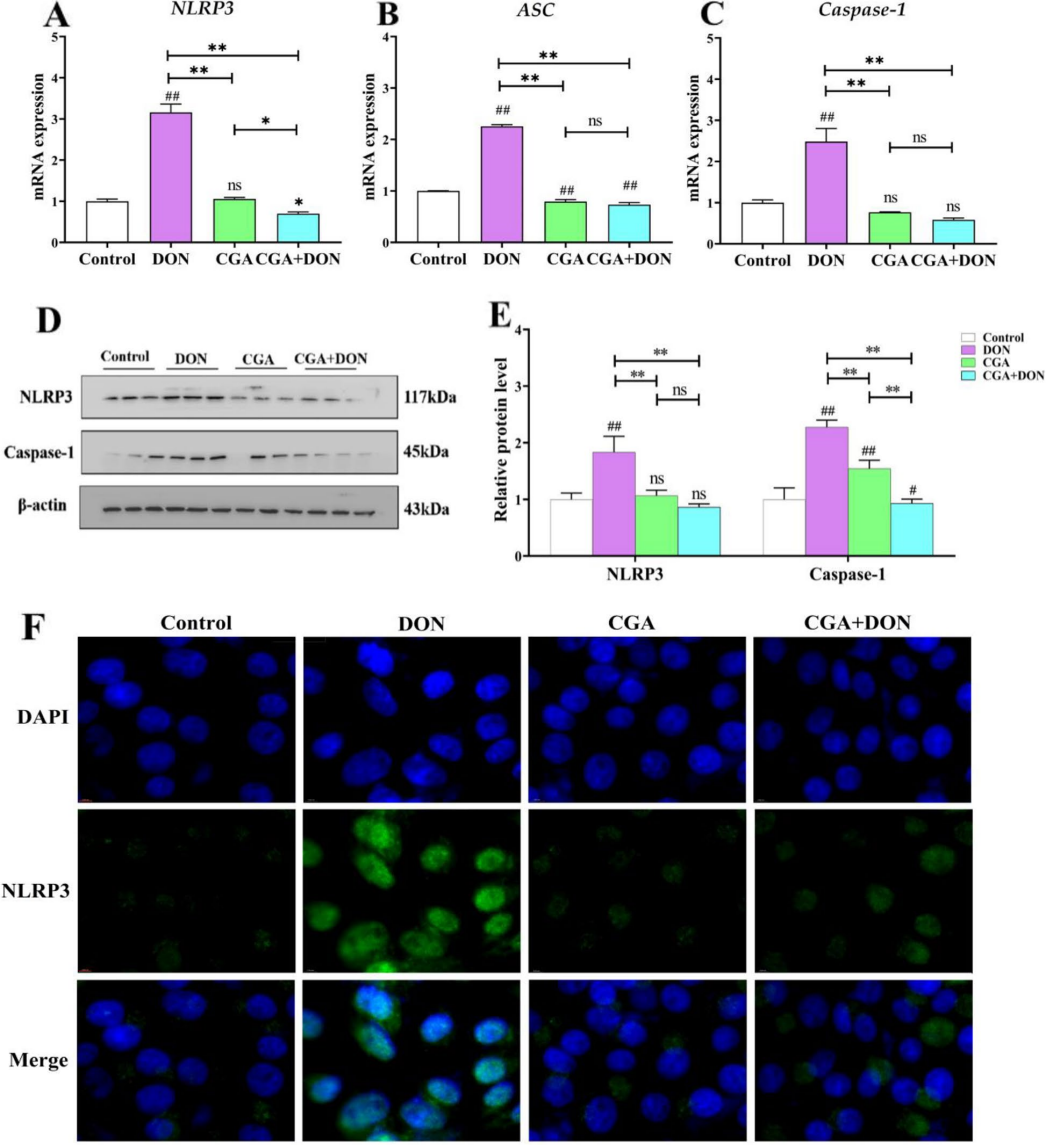
Effects of CGA and DON on activation signaling factors related to pyroptosis. mRNA levels of (**A**) *NLRP3*, (**B**) *ASC*, and (**C**) *Caspase-1* (mean ± SD, *n* = 6). **D** Protein bands and expression levels of (**E**) NLRP3 and Caspase-1 (mean ± SD, *n* = 3). **F** Fluorescence images of NLRP3 were observed by confocal immunofluorescence (*n* = 3). Compared with the control group, ## and # represent *P* < 0.01 and* P* < 0.05 respectively, and compared with the other group, ** and * represent *P* < 0.01 and* P* < 0.05 respectively. ns indicates *P* > 0.05

qRT‒PCR data (Fig. 9A–C) showed that DON exposure significantly increase the mRNA expression levels of *caspase-1*, *ASC* and *NLRP3* compared with that in the control group (*P* < 0.01). Meanwhile, the mRNA expression levels of *caspase-1*, *ASC* and *NLRP3* in the CGA + DON group were higher than those in the DON group (*P* < 0.01). Western bolt results (Fig. 9D and E) displayed that the protein expression levels of caspase-1 (*P* < 0.01) and NLRP3 (*P* < 0.01) significantly increased in the DON group compared with that in the control group, whereas in contrast to the DON group, the protein expression levels of caspase-1 (*P* < 0.01) and NLRP3 (*P* < 0.01) significantly decrease in the CGA + DON group (*P* < 0.01), indicating that the activation of the NLRP3 inflammasome and caspase-1 induced by DON was inhibited by CGA. The IF images illustrated that the fluorescence intensity of NLRP3 in the DON group was significantly stronger than that in the other three groups (Fig. 9F), while the fluorescence intensity in the CGA + DON group was significantly lighter compared with that in the DON group.

## Discussion

### DON induces IPEC-J2 cell pyroptosis

DON is recognized as one of the most prevalent mycotoxins worldwide [4, 5]. The intestinal barrier plays a crucial role in safeguarding the intestinal tract against infections and ensuring the maintenance of body homeostasis [32]. Pigs are particularly susceptible to DON, and IPEC-J2 cells are commonly employed for studying the impact of nutrients or toxins on intestinal barrier function [33]. In this study, IPEC-J2 cells were exposed to a concentration gradient of six different doses of DON ranging from 500 to 3,000 ng/mL, and the findings demonstrated a corresponding decrease in cell viability and an increase in mortality with increasing concentration and incubation time, which is consistent with previous studies [34, 35].

Pyroptosis is an immunoinflammatory response that plays a crucial role in the occurrence and progression of various diseases [14, 36, 37]. Although previous studies have demonstrated that exposure to DON from 1,000 to 2,000 ng/mL can induce apoptosis and autophagy [22, 38, 39], few studies have been conducted on the impact of DON on pyroptosis, particularly regarding its underlying mechanisms and activation pathways. Pyroptosis is characterized by inflammasome formation and the subsequent release of IL-1β and IL-18 as well as prominent morphological features that include chromosomal DNA degradation, loss of membrane integrity, and cytoplasmic material release [11].

In this study, IPEC-J2 cells treated with DON exhibited typical pyroptosis characteristics, including lysosomal damage, the formation of pores in the cell membrane, the release of positive intracellular ions and the inflammatory factors IL-1β and IL-18, and the activation of caspase-1 and caspase-4. The extent of cellular damage increased with increasing concentrations of DON. Conversely, significant decreases in the levels of LDH and pro-IL-1β were observed at a concentration of 3,000 ng/mL compared with 2,500 ng/mL. This discrepancy could be attributed to excessive cell mortality, which may have interfered with the final experimental results.

### CGA inhibits DON-induced pyroptosis through the NF-κB/NRPR3/caspase-1 signaling pathway

Inhibiting the activation and development of pyroptosis could reduce the pathological damage caused by immune inflammation [16, 40, 41]. Consequently, intervening in the pyroptosis pathway mediated by the NLRP3 inflammasome may serve as a new strategy and therapeutic target for alleviating DON toxicity. The polyphenol CGA is present in various plants and foods. Numerous studies have demonstrated the diverse biological effects of CGA, including antioxidant, antibacterial, anti-inflammatory, hepatoprotective, and anticancer properties [42–44]. Previous research in our laboratory indicated that the addition of CGA augments the anti-inflammatory and antioxidant capabilities of rabbit intestines [45]. In this study, IPEC-J2 cells were pretreated with a gradient of 10–100 μmol/L CGA for 4 h, followed by induction with 2,500 ng/mL DON. The protective effect on the cells was positively correlated with the amount of added CGA, and the strongest protection against cell damage induced by DON was observed with 80 μmol/L CGA. However, increasing the CGA concentration to 100 μmol/L resulted in a decreasing trend in cell viability. These findings suggest a biphasic trend in the reduction in DON-induced cell damage caused by CGA, characterized by an initial increase followed by a subsequent decrease.

Previous studies have demonstrated a close association between the cytotoxic mechanism induced by DON and the Nrf2/HO-1, MAPK and NF-κB signaling pathways [26, 46]. Oxidative stress can trigger apoptosis, and Nrf2 effectively counteracts the production of ROS, thereby maintaining the intracellular redox balance [47]. Furthermore, both ROS production and activation of the MAPK signaling pathway can stimulate the NF-κB pathway [22], and NF-κB activation by ROS depends on the actual level of redox imbalance. These findings suggest that CGA significantly inhibits the oxidative stress, apoptosis and activation of NF-κB induced by DON.

Pyroptosis can be triggered through either the classical pathway involving caspase-1 activation or the nonclassical pathway involving caspase-11/4/5 activation [48]. This study revealed that CGA notably inhibited DON-induced activation of both caspase-1 and NLRP3. The activation of caspase-1 contributes to the hydrolysis of GSDMD into N-terminal and C-terminal fragments, after which the N-term forms pores on the cell membrane through oligomerization, leading to the efflux of intracellular IL-1β and IL-18 and ultimately inducing pyroptosis. When apoptosis or pyroptosis occurs, the integrity of the cell membrane is compromised, resulting in the release of LDH from the cytoplasm into the culture medium. Consequently, LDH activity typically serves as an indirect indicator for evaluating cell membrane integrity [46]. In this study, pretreatment with CGA significantly alleviated DON-induced damage to the cellular membrane and overflow of positive ions, thereby mitigating the imbalance in intracellular cation levels as well as the immune inflammatory response mediated by IL-1β and IL-18.

Studies have proposed a dual-signal model associated with the process of pyroptosis, which involves both initiating and activating signals of the NLRP3 inflammasome [16]. Several studies have demonstrated that DON can induce pyroptosis in a variety of cell lines, but whether DON induces pyroptosis through a dual signaling pathway is unclear. Therefore, this study investigated the role of initiation and activation signaling factors in DON-induced pyroptosis. In this study, following exposure of IPEC-J2 cells to 2,500 ng/mL DON for 24 h, the initiating signaling factors, including *TNF*, *MDP*, *NOD2* and *TLR4*, were upregulated, thereby promoting the massive expression of NF-κB. DON can also directly induce the overproduction of ROS and further activate NF-κB. Under the joint stimulation of NF-κB and oxidative stress, the expression of the activation signaling factors NLRP3, ASC and pro-caspase-1 was upregulated, thereby promoting the assembly and activation of the NLRP3 inflammasome complex and subsequently inducing caspase-1 activation. On the one hand, activated caspase-1 promotes the cleavage of pro-IL-1β and pro-IL-18 into their respective active forms, IL-1β and IL-18. On the other hand, it promotes the cutting of GSDMD into the GSDMD-N domain and the formation of membrane pores, resulting in the overflow of large amounts of IL-1β and IL-18 that disrupt the osmotic potential, cause cellular swelling, and ultimately induce pyroptosis.

**Fig. 10:**
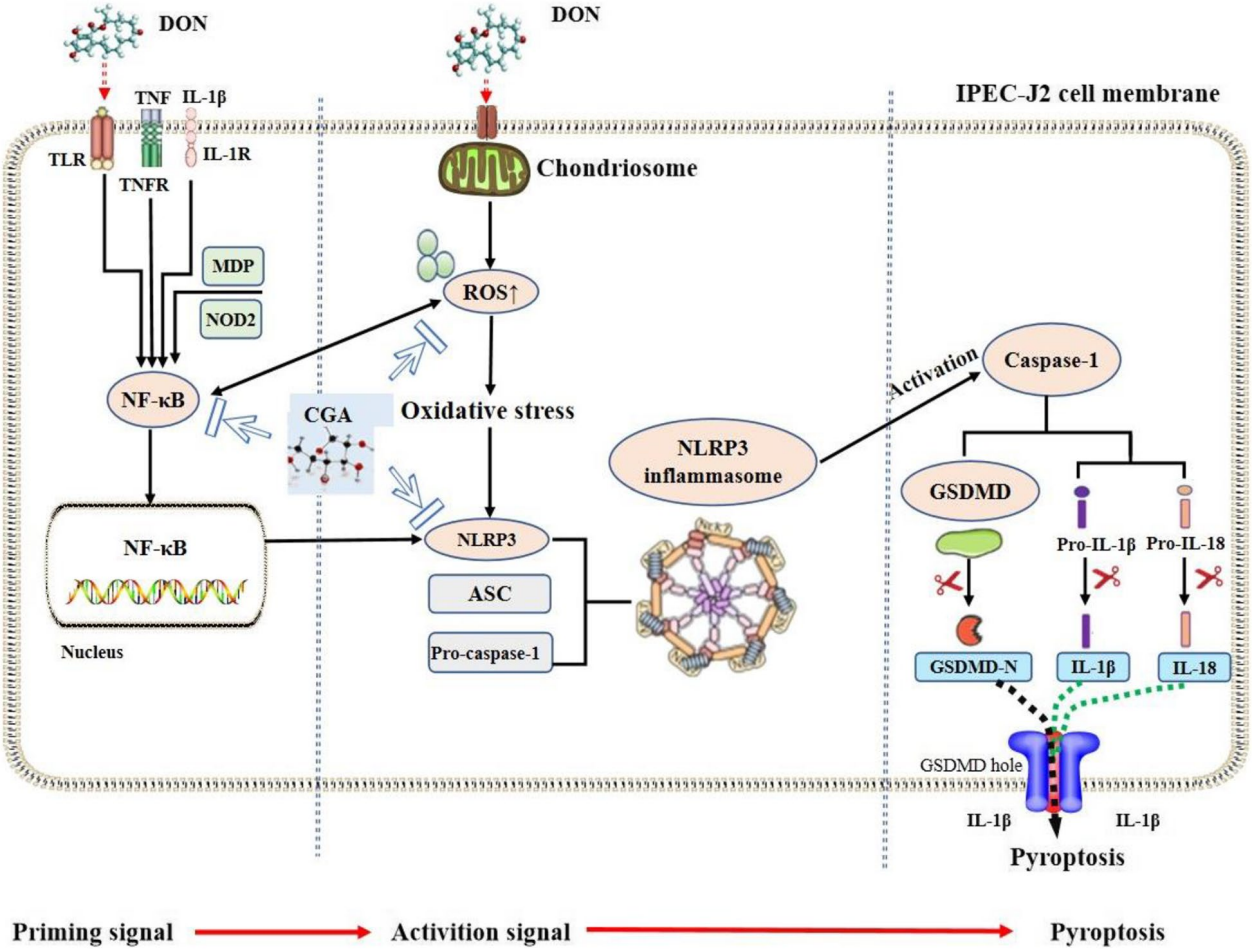
Schematic diagram illustrating how CGA alleviates DON-induced pyroptosis by inhibiting dual-signaling pathway

The findings of this study suggest that exposure to DON can activate the NLRP3 inflammasome and induce pyroptosis through dual signaling. However, intervention with 80 μmol/L CGA alleviated DON-induced pyroptosis by inhibiting both the initiating and activating signals, particularly targeting the NF-κB/NLRP3/caspase-1 pathway. A schematic diagram illustrating the effects of CGA on DON-induced pyroptosis is presented in Fig. 10.

## Conclusions

In summary, pretreatment with 80 μmol/L CGA for 4 h effectively alleviated pyroptosis in IPEC-J2 cells induced by 2,500 ng/mL of DON through inhibiting both initiating and activating signals, specifically targeting the NF-κB/NLRP3/capase-1 pathway. Based on the above findings, CGA exhibits potential as a promising feed additive for mitigating intestinal damages induced by DON in animals.

## Supplementary Information

Additional file 1: Fig. S1. Diagram illustrating the dual-signaling pattern of the initiation and activation of the NLRP3 inflammasome.

Additional file 2: Table S1. Information of main chemical reagents used in this study.

Additional file 3: Table S2. Information of detection kits used in this study.

Additional file 4: Table S3. The prime sequence synthesized by for qRT-PCR analysis used in this study.

Additional file 5: Table S4. Information of antibodies used in Western blotting and Immunofluorescence.

## Data Availability

The data used to support the findings of this study are available from the corresponding author upon reasonable request.

## Abbreviations

AO: Acridine orange
ASC: Apoptosis-associated speck-like protein containing a caspase recruitment domain
CGA: Chlorogenic acid
DCFH-DA: Dichlorofluorescin diacetate
DMSO: Dimethyl sulfoxide
DON: Deoxynivalenol
EB: Ethidium bromide
GSDMD: Gasdermin D
IF: Immunofluorescence
IL: Interleukin
IPEC-J2: Intestinal porcine epithelial cell line-J2
LDH: Lactate dehydrogenase
LPS: Lipopolysaccharide
LTR: Lyso-Tracker Red
MDP: Muramyl dipeptide
NF-κB: Nuclear factor kappa-B
NLRP3: Nucleotide oligomerization domain-like receptor thermal protein domain-associated protein 3
NOD 2: Nucleotide oligomerization domain-like receptor-2
PMSF: Phenylmethylsulfonyl fluoride
ROS: Reactive oxygen species
SEM: Scanning electron microscope
TLR4: Toll-like receptors 4
TNF: Tumor necrosis factor
TUNEL: Terminal deoxynucleotidyl transferase–mediated dUTP-biotin nick end labeling assay
