# Supplementary material for: Chlorogenic acid alleviates IPEC-J2 pyroptosis induced by deoxynivalenol by inhibiting activation of the NF-κB/NLRP3/caspase-1 pathway
Source: J Anim Sci Biotechnol. 2024 Dec 2;15:159. doi: 10.1186/s40104-024-01119-z (PMC11610088; doi:10.1186/s40104-024-01119-z)
Supplement: Supplementary file 1 — Additional file 1: Fig. S1. Diagram illustrating the dual-signaling pattern of the initiation and activation of the NLRP3 inflammasome. [file 40104_2024_1119_MOESM1_ESM.doc]

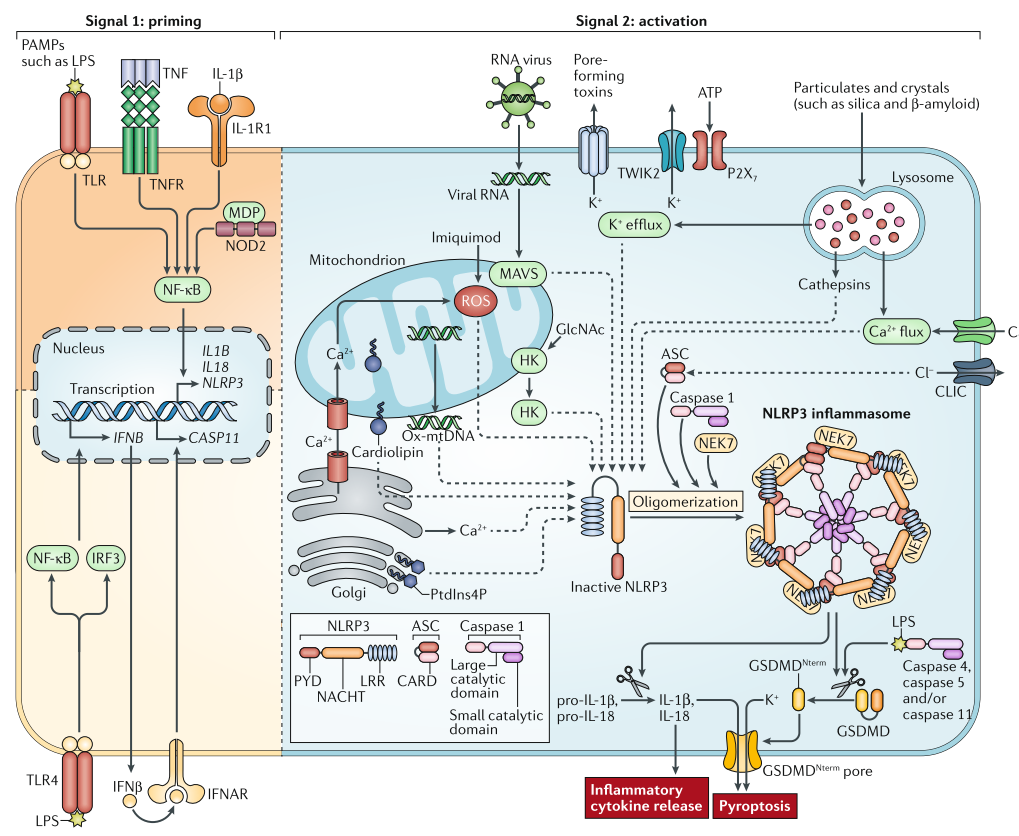


**Figure S1.** Diagram illustrating the dual-signaling pattern of the initiation and activation of the NLRP3 inflammasome [16].
