# Supplementary material for: Chlorogenic acid alleviates IPEC-J2 pyroptosis induced by deoxynivalenol by inhibiting activation of the NF-κB/NLRP3/caspase-1 pathway
Source: J Anim Sci Biotechnol. 2024 Dec 2;15:159. doi: 10.1186/s40104-024-01119-z (PMC11610088; doi:10.1186/s40104-024-01119-z)
Supplement: Supplementary file 2 — Additional file 2: Table S1. Information of main chemical reagents used in this study. [file 40104_2024_1119_MOESM2_ESM.doc]

**Table S1. Information of main chemical reagents used in this study**

| Reagents | Manufacturer | Location |
| --- | --- | --- |
| 0.25% pancreatin with or without EDTA | Solarbio Biotechnology Co., Ltd. | Beijing, China |
| 4% paraformaldehyde |
| Dimethyl sulfoxide (DMSO) |
| Penicillin (10000 U/mL) - streptomycin (10 mg/mL) mixture |
| Protease inhibitor mixture (100 x) |
| Triton X- 100 |
| BSA、DAPI dying solution | Beyotime Biotechnology Co., Ltd. | Shanghai, China |
| Phenylmethylsulfonyl fluoride (PMSF) |
| RIPA lysis buffer |
| DMEM high-glucose culture medium | Pricella Biotechnology Co., Ltd | Wuhan, China |
| Fetal bovine serum |
| Polyvinylidene fluoride (PVDF) membrane | EMD Millipore Corpration | Shanghai, China |
| TBST | New Cell& Molecular Biotech Co., Ltd | Suzhou, China |
| Trizol reagents | Thermo fisher scientific | California, USA |
