# Supplementary material for: Chlorogenic acid alleviates IPEC-J2 pyroptosis induced by deoxynivalenol by inhibiting activation of the NF-κB/NLRP3/caspase-1 pathway
Source: J Anim Sci Biotechnol. 2024 Dec 2;15:159. doi: 10.1186/s40104-024-01119-z (PMC11610088; doi:10.1186/s40104-024-01119-z)
Supplement: Supplementary file 3 — Additional file 3: Table S2. Information of detection kits used in this study. [file 40104_2024_1119_MOESM3_ESM.doc]

**Table S2. I**nformation of detection kits used in this study

| Detection kit | Manufacturer | Location |
| --- | --- | --- |
| CCK-8 assay kit | Solarbio Biotechnology Co., Ltd. | Beijing, China |
| AO/EB dual-dye kit | Yuanye Bio-Technology Co., Ltd. | Shanghai, China |
| DCFH-DA probe | MedChemexpress Biotech Co., Ltd. | New Jersey, USA |
| Detection assay kits for Ca^2+^ and K^+^ | Beyotime Biotechnology Co., Ltd. | Shanghai, China |
| EasyScript® One-Step gDNA Removal and cDNA Synthesis Super Mix | TransGen Biotechnology Co., Ltd. | Beijing, China |
| ELISA assay kits | Meimian Industrial Inc. | Jiangsu, China |
| Enzyme activity detection kits for Caspase-1/4 | Beyotime Biotechnology Co., Ltd. | Shanghai, China |
| LDH Cytotoxicity Assay Kit | Beyotime Biotechnology Co., Ltd. | Shanghai, China |
| Low background luminescence ECL detection kit | Beyotime Biotechnology Co., Ltd. | Shanghai, China |
| Lyso-Tracker Red | Beyotime Biotechnology Co., Ltd. | Shanghai, China |
| MTT Cell Proliferation and Cytotoxicity Assay Kit | Solarbio Biotechnology Co., Ltd. | Beijing, China |
| PrimeScript®RT Reagent Kit | TaKaRa Biotechnology Co., Ltd. | Shiga, Japan |
| SYBR Green Premix Pro Taq HS qPCR Kit | Success Biotechnology Co., Ltd | Shandong, China |
| TUNEL apoptosis detection kit | Beyotime Biotechnology Co., Ltd. | Shanghai, China |
