# Supplementary material for: Chlorogenic acid alleviates IPEC-J2 pyroptosis induced by deoxynivalenol by inhibiting activation of the NF-κB/NLRP3/caspase-1 pathway
Source: J Anim Sci Biotechnol. 2024 Dec 2;15:159. doi: 10.1186/s40104-024-01119-z (PMC11610088; doi:10.1186/s40104-024-01119-z)
Supplement: Supplementary file 4 — Additional file 4: Table S3. The prime sequence synthesized by for qRT-PCR analysis used in this study. [file 40104_2024_1119_MOESM4_ESM.doc]

**Table S3. The prime sequence synthesized by for qRT-PCR analysis used in this study.**

| **Gene** | **Forward primer (5´ to 3´ direction)** | **Reverse primer (5´ to 3´ direction)** | **length (bp)** | **Gene ID** | **GenBank No.** |
| --- | --- | --- | --- | --- | --- |
| *ASC* | *CGTGACATCGGCATGAAGGAG* | *GTGCTGGTTTGTTGTCTGCTTTC* | 118 | 100522011 | XM_003124468.5 |
| *Caspase-1* | *ACTCTCCACAGGTTCACAATCT* | *TCTGAAGACGCAGGCTTAACT* | 120 | 397319 | NM_214162.1 |
| *GSDMD* | *CGGAAGAAGACGGTCACCATC* | *ATGTCCCAGTCAGAACCAATCAC* | 83 | 100515607 | XM_021090504.1 |
| *IL-1β* | *AAGTGGTGTTCTGCATGAGCTTT* | *CAGGGTGGGCGTGTTATCTT* | 125 | 397122 | NM_214055.1 |
| *IL-18* | *CCAGGGACATCAAGCCGTGTT* | *TGGTTACTGCCAGACCTCTAGTGA* | 122 | 397057 | NM_213997.1 |
| *MDP* | *GTCACGCACTTTGAGAGGTTG* | *GGACATGAACACAGGCAACAC* | 124 | 100520440 | XM_003128546.4 |
| *NF-κB* | *ATGTGGGACCAGCAAAGGTT* | *CACCATGTCCTTGGGTCCAG* | 134 | 4790 | NM_001165412.2 |
| *NLRP3* | *AGGAGGAGGAAGAGGAGATAGAG* | *GGACTGAGAAGATGCCACTACA* | 144 | 100514823 | NM_001256770.2 |
| *NOD2* | *TGTGAAGGCGAATGGGTTGG* | *GTACTTCTTACAGGCAGCATCTTC* | 100 | 100125838 | NM_001105295.1 |
| *TLR4* | *TGCAGAAGTTGGAGAAGTCCC* | *CCTCCCACTCCAGGTAGGTAT* | 84 | 399541 | NM_001113039.2 |
| *TNF* | *ACCACGCTCTTCTGCCTACTG* | *GACGGGCTTATCTGAGGTTTGAGA* | 132 | 397086 | NM_214022.1 |
| *GAPDH* | *ATGGTGAAGGTCGGAGTGAAC* | *GTGGGTGGAATCATACTGGAACA* | 153 | 396823 | NM_001206359.1 |
