# Supplementary material for: Chlorogenic acid alleviates IPEC-J2 pyroptosis induced by deoxynivalenol by inhibiting activation of the NF-κB/NLRP3/caspase-1 pathway
Source: J Anim Sci Biotechnol. 2024 Dec 2;15:159. doi: 10.1186/s40104-024-01119-z (PMC11610088; doi:10.1186/s40104-024-01119-z)
Supplement: Supplementary file 5 — Additional file 5: Table S4. Information of antibodies used in Western blotting and Immunofluorescence. [file 40104_2024_1119_MOESM5_ESM.doc]

### Table S4. Information of antibodies used in western blotting and Immunofluorescence

| Antibody | Source | Dilution | Information |
| --- | --- | --- | --- |
| Primary antibody | | | |
| IL-1β Polyclonal Antibody | Rabbit | 1:1000 | AF7209 (Beyotime, Shanghai, China) |
| IL-18 Polyclonal Antibody | Rabbit | 1:1000 | AF7266 (Beyotime, Shanghai, China) |
| Caspase-1 Polyclonal Antibody | Rabbit | 1:1000 | 22915-1-AP (Proteintech, Wuhan, China) |
| NF-κB Polyclonal Antibody | Rabbit | 1:1000 | 10745-1-AP (Proteintech, Wuhan, China) |
| NLRP3 Polyclonal Antibody | Rabbit | 1:1000 | 27458-1-AP (Proteintech, Wuhan, China) |
| β-actin Polyclonal Antibody | Rabbit | 1:5000 | ab8227 (Abcam, Cambridgeshire, UK) |
| Secondary antibody | | | |
| HRP Antibody | Goat anti rabbit | 1:5000 | RGAR001 (Proteintech, Wuhan, China) |
